# Influence of Neutral Axis on Performance Stability of Flexible Antennas for ISM Band Wearable Applications

**DOI:** 10.3390/s26154876

**Published:** 2026-08-02

**Authors:** Dipon Saha, Nursabirah Jamel, Illani Mohd Nawi

**Affiliations:** 1 Department of Electrical and Electronic Engineering, Universiti Teknologi PETRONAS, Seri Iskandar 32610, Perak, Malaysia; 2 Department of Electrical and Electronic Engineering, Daffodil International University, Dhaka 1216, Bangladesh; 3 Faculty of Electrical Engineering & Technology, Universiti Malaysia Perlis, Arau 02600, Perlis, Malaysia; 4 Centre of Excellence for Micro System Technology (MiCTEC), Universiti Malaysia Perlis, Arau 02600, Perlis, Malaysia

**Keywords:** antenna bending, CPW patch, encapsulation, neutral axis, wearable antenna

## Abstract

Wearable textile antennas are commonly used in various applications nowadays, such as watches and clothes; however, they are still subjected to degradation in the reflection coefficient and shift in the resonance frequency due to bending or twisting. This study presents a neutral axis-based encapsulation technique to improve the electromagnetic stability of flexible antennas. A 2.45 GHz CPW patch antenna is designed using a polyester substrate, silver conductive layer, and TPU as encapsulation for the encapsulated antenna. By aligning the conductive layer with the neutral axis, the proposed antenna achieved stable resonance characteristics under both convex and concave bending conditions. In contrast, the non-encapsulated antenna exhibited significant resonance degradation under deformation. Comparative analysis further showed that displacing the conductive layer away from the neutral axis increased the maximum resonance frequency shift to 8.38%, whereas the neutral axis aligned configuration maintained a substantially lower shift of only 3.55%. These results validate the effectiveness of neutral axis engineering for enhancing the deformation resilience of flexible wearable antennas.

## 1. Introduction

Wearable textile antennas have generated a vast amount of interest among researchers in the fields of healthcare, sports, security, and military applications. This resulted in the growth of a wide variety of wireless body-centric technologies that are expanding quickly [1]. By integrating a communication system into the notion of clothing, it is possible to provide a wearable tracking and navigation-based application, improving people’s quality of life. Through the development of wearable textile antennas, body-centric wireless communication establishes a link between wearable electronics and the surrounding environment [2], while providing flexibility, compactness, washability, softness, and comfort that textile antennas provide to the wearer. There are several different types of antennas that can be used in various wearable applications. Some common types of wearable antennas are patch antennas, which are flat antennas typically printed on a flexible substrate and can be attached to the body or integrated into wearable devices. They are often used for applications that require moderate to high gain and operate in specific frequency bands. Dipole antennas are simple antennas consisting of two conductive elements and can be designed as wire-like structures that can be integrated into wearable devices or can be incorporated into clothing as fabric-based antennas.

The constraints of size, form factor, and placement on the body or wearable devices introduce challenges in the development process of conventional wearable antennas. For ensuring reliable wireless functionality in wearable devices, parameters including operational frequency, bandwidth, and radiation pattern need to be properly taken into account. Flexible antennas encounter a range of challenges, particularly related to deformations caused by stretching, bending, and twisting that occur during everyday use [3]. Many studies have optimized flexible antennas for application-specific bending conditions. For instance, implantable antennas are designed and tested under controlled deformations that simulate biological tissue movement [4,5], while textile-based sensing antennas are often evaluated for predefined bending angles to match their expected wear conditions [6,7]. In real-world applications, wearable antennas experience continuous and unpredictable deformations, and most of the flexible antennas fail to sustain performance across arbitrary bending and twisting conditions [8]. To mitigate these limitations, the antenna structure can be engineered to minimize the influence of externally induced mechanical stress and strain on the electromagnetic characteristics of the antenna. Positioning the radiating elements near the neutral axis of the multilayer structure can reduce bending or deformation induced strain in the conductive layer of the antenna.

**Figure 1:**
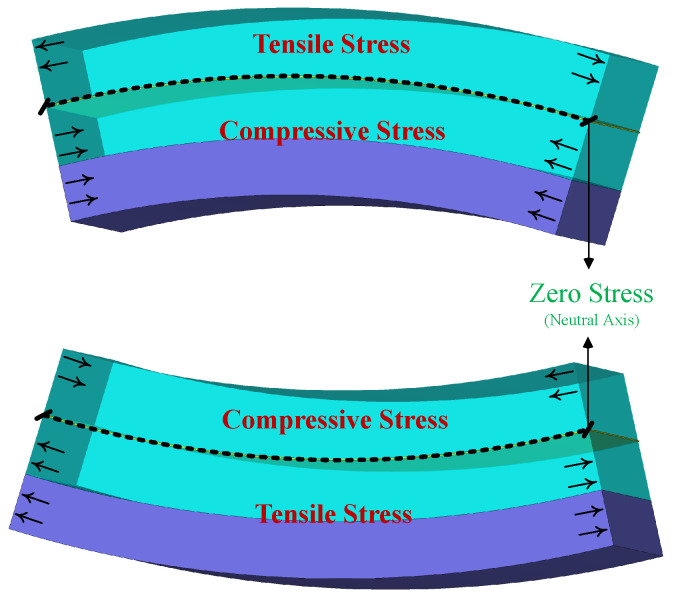
Illustration of stress distribution in a multilayer flexible structure under bending deformation [9].

The neutral axis refers to the plane where the stress is zero, where there is neither tension nor compression due to bending or deformation [9]. During bending, layers above the neutral axis undergo tensile stress, whereas layers below experience compressive stress as presented in Figure 1. By aligning the conductive traces and critical radiating components close to the neutral axis, the mechanical deformation experienced by the antenna can be significantly reduced, resulting in stable radiation performance, resonance response, and impedance matching while in dynamic bending conditions. In ref. [10], the authors empirically demonstrate that electrical interconnects tend to be more robust and reliable when placed on or near the neutral axis of the e-textile, as the internal stress is reduced. In symmetric and isotropic beams, the neutral axis is at the geometrical centroid. As demonstrated in [11], if the beam is asymmetrical, force equilibrium needs to be used to identify the neutral axis’s location. However, the neutral axis may not be close to the centroid in woven and knitted fabrics [12]. A study [13] calculated the bending rigidness of multilayered textiles to determine the neutral axis’s relative position in fabrics. Furthermore, by analyzing the electrical resistivity variation in the printed strain gauge, the authors in [14] experimentally determined the neutral axis of screen-printed e-textiles. In this experiment, by adjusting the screen-printed material layer thicknesses, the strain gauges have been placed at various heights within the printed structure. The strain gauge positioning, which indicated the least variation in electrical resistance while bent, was taken into account to determine the neutral axis location. The position of the neutral axis is particularly critical in wearable antennas, since when the body moves, the supporting textile substrate is constantly bent, stretched, and undergoes various mechanical deformations.

In this study, a 2.45 GHz coplanar waveguide (CPW) patch antenna is designed and simulated as the reference antenna structure. The proposed antenna is subsequently encapsulated using a flexible material in which the conductive layer is positioned along the neutral axis of the multilayer structure. Furthermore, an additional configuration is investigated in which the conductive layer is intentionally displaced away from the neutral axis to evaluate the performance of the antenna while considering the neutral axis theory. The following sections present the antenna design and simulation methodology, multilayer encapsulation structure, and bending performance analysis, providing systematic evidence for the effectiveness of neutral axis-based flexible antenna engineering.

This paper is organized as follows, with the introduction and background presented in Section 1, the discussion flows into the modeling and simulation of the proposed antenna, including the simulation configuration, antenna materials, design of the CPW antenna, and the determination of the neutral axis and encapsulation presented in Section 2. Section 3 discusses the results, including the reflection coefficient of the CPW antenna and its behavior under bending, the reflection coefficient of the encapsulated CPW antenna and its behavior under bending, the effect of the neutral axis, and the radiation pattern analysis of the CPW and encapsulated CPW antennas under flat and bent conditions. Section 4 presents a comparative analysis with recent state-of-the-art literature. Finally, Section 5 concludes the paper.

## 2. Modeling and Simulation

The modeling and simulation for this research were conducted using the Computer Simulation Technology (CST) Microwave Studio (v2019). Simulations have been performed on two different antenna models. First, a CPW antenna was designed and simulated without consideration of the neutral axis, which serves as the base model or reference model of this study. Subsequently, the CPW antenna was encapsulated within a multilayer structure and re-simulated to analyze the effects of neutral axis bending on the performance of the flexible antenna. Furthermore, to isolate and examine the specific effect of neutral axis positioning, additional encapsulated configurations have also been analyzed in which the conductive layer was intentionally positioned away from the neutral axis.

Antenna simulation involves Maxwell’s equations, which describe how electric and magnetic fields propagate and interact [15,16]. CST MWS utilizes finite integration technique (FIT) solver, an exceptionally versatile method that allows the representation of Maxwell’s equations within a grid space. FIT can solve for both the time domain and the frequency domain. Moreover, FIT is not limited to any specific grid type, enhancing its applicability across various types of electromagnetic problems [17]. Maxwell’s equations in differential form are as follows,

(1) $$\begin{array}{cc} \nabla \cdot E & =\frac{\rho }{\epsilon_{\circ }} \end{array}$$

(2) $$\begin{array}{cc} \nabla \cdot B & =0 \end{array}$$

(3) $$\begin{array}{cc} \nabla \times E & =\frac{\partial B}{\partial t} \end{array}$$

(4) $$\begin{array}{cc} \nabla \times B & =\mu_{\circ }J+\mu_{\circ }\epsilon_{\circ }\frac{\partial E}{\partial t} \end{array}$$

where $\epsilon_{\circ }$ stands for free space permittivity, ρ for electric charge density, *E* for electric field, *B* for magnetic flux density, *t* for time, $\mu_{\circ }$ for free space permeability, and *J* for current density.

The electric field wave equation is given by,

(5) $$\nabla^{2}E-\mu_{\circ }\epsilon_{\circ }\frac{\partial^{2}E}{\partial t^{2}}=-\mu_{\circ }\epsilon_{\circ }\frac{\partial J}{\partial t}$$

The magnetic field wave equation is given by,

(6) $$\nabla^{2}B-\mu_{\circ }\epsilon_{\circ }\frac{\partial^{2}B}{\partial t^{2}}=\nabla \times J$$

In contrast to the majority of numerical algorithms, FIT discretizes the subsequent integral representation of Maxwell’s equations for a variety of electromagnetic issues, spanning static field computations to high-frequency tasks in either the time or frequency domain [18,19,20],

(7) $$\begin{array}{cc} \oint_{\partial A}\vec{E}\cdot d\vec{s} & =-\int_{A}\frac{\partial \vec{B}}{\partial t}\cdot d\vec{A} \end{array}$$

(8) $$\begin{array}{cc} \oint_{\partial A}\vec{H}\cdot d\vec{s} & =\int_{A}\left(\frac{\partial \vec{D}}{\partial t}+\vec{J}\right)\cdot d\vec{A} \end{array}$$

(9) $$\begin{array}{cc} \oint_{\partial V}\vec{D}\cdot d\vec{A} & =\int_{V}\rho \;dV \end{array}$$

(10) $$\begin{array}{cc} \oint_{\partial V}\vec{B}\cdot d\vec{A} & =0 \end{array}$$

Simulation configuration, selection of materials, antenna modeling, and determination of the neutral axis are discussed in detail in the following Section 2.1, Section 2.2, Section 2.3 and Section 2.4.

### 2.1. Simulation Configuration

The antenna was simulated using CST Studio Suite 2019 with the Transient (Time-Domain) solver, which was automatically recommended by the project template wizard for waveguide antenna applications. The transient solver computes the reflection coefficient via the FIT with Perfect Boundary Approximation (PBA), which is well suited for analyzing complex antenna structures as the PBA-based hexahedral mesh accommodates fine geometric features without requiring explicit triangulation [21]. The simulation frequency range was set from 1.9 GHz to 3 GHz, covering the target ISM band with sufficient margin. All six boundary faces were assigned Expanded Open boundary conditions with a Convolution PML (Perfectly Matched Layer) of four layers, with automatically determined depths ranging from 14.0 mm to 16 mm, effectively emulating an unbounded free-space environment and minimizing spurious reflections from the domain boundaries. No symmetry conditions were applied in order to preserve the full generality of the electromagnetic field solution. A waveguide port with a reference impedance of 50 Ω was used to excite the antenna, and the solver accuracy was set to −60 dB to ensure a well-converged time-domain solution.

To verify numerical convergence with respect to mesh discretization, a systematic mesh convergence study was conducted for all antenna configurations under both normal and bending conditions, as summarized in Table 1. For each configuration, multiple simulation runs were performed with progressively decreasing mesh cell counts, and the resonant frequency was recorded at each level. The step deviation between successive mesh refinements was computed to quantify the sensitivity of the solution to mesh density. For the unencapsulated CPW antenna, the resonant frequency varied from 2.38 GHz at 69,741 cells to 2.45 GHz at 139,536 cells, with step deviations decreasing from 0.030 GHz to 0.015 GHz, indicating a converging trend. Under bending conditions, the CPW antenna exhibited resonant frequencies ranging from 2.455 GHz to 2.505 GHz across the mesh range of 435,200 to 567,000 cells, with a minimum step deviation of 0.010 GHz at the finest mesh. For the encapsulated CPW antenna, the resonant frequency stabilized at 2.395 GHz for the two finest mesh runs (237,600 and 255,000 cells), yielding a step deviation of 0.000 GHz, which provides strong evidence of mesh-independent convergence. Similarly, the encapsulated CPW under bending conditions showed consistent convergence, with the frequency stabilizing near 2.475–2.48 GHz at the finer mesh levels. Based on these results, the finest mesh configuration for each case was adopted for all subsequent analyses reported in this study.

### 2.2. Antenna Materials

Material selection for the antenna is very crucial, as this greatly affects the performance of the designed antenna. The performance of wearable antennas is largely determined by the material of two key components, the substrate, which uses nonconductive material and the radiating element, which uses conductive materials [22,23]. In this study, two additional layers have been used, which are the encapsulation layer and the interface layer. These layers are important, especially for wearable applications, as they will minimize the surface roughness and also minimize moisture absorption from the textile [24]. Three different materials have been utilized in this study, which are silver, polyester and thermoplastic polyurethane (TPU). Silver is selected as the conductive material for the antenna due to its high electrical conductivity of $6.301\times 10^{7}$ S/m [25], which ensures efficient signal transmission. Polyester is used as the substrate because of its low conductivity and mechanical flexibility. Polyester is more suitable for flexible electronic applications, having a dielectric constant of 1.5 and a Young’s modulus of 3.25 kN/mm^2^ [3]. TPU serves as the material for both the encapsulation layer and the interface layer, providing protection and mechanical support. TPU features a Young’s modulus of 0.033 kN/mm^2^ [26], which allows TPU to accommodate deformation while maintaining performance. Table 2 presents the detailed properties of the materials used in this study.

### 2.3. Design of the CPW Antenna

**Figure 2:**
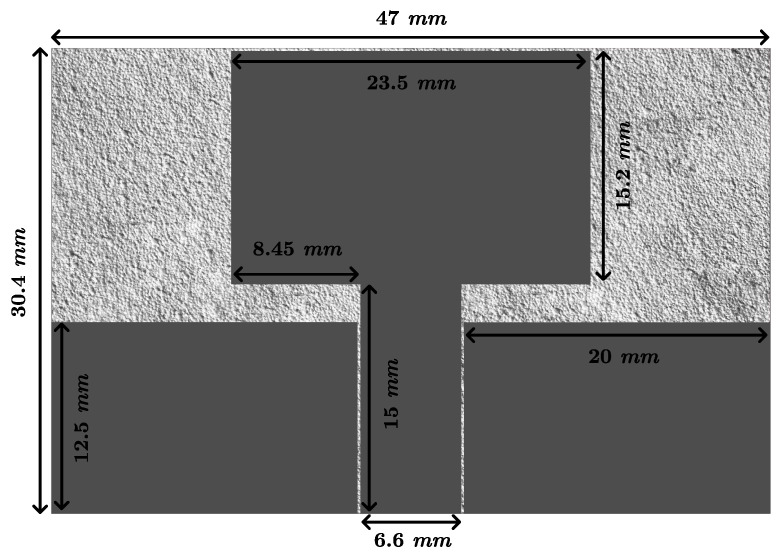
Dimensions of the CPW patch antenna.

In this study, the design and analysis of a CPW antenna are presented, which has been used to investigate the durability and effectiveness of the antenna in bent conditions. Polyester substrate with a relative permittivity $\epsilon_{r}$ of 1.5 has been used to model the antenna. Polyester is chosen for the substrate layer since polyester is flexible and appropriate for wearable and conformal uses [27], and the substrate utilized has a thickness of 1.5 mm and dimensions of 30.4 mm × 47 mm. The ground plane and the radiating patch of the CPW antenna are constructed using a highly conductive silver [28] with a thickness of 0.03 mm. A CPW transmission line that is 15 mm long and 6.6 mm wide feeds the patch, which is 15.2 mm × 23.5 mm. The ground planes, positioned symmetrically on either side of the transmission line, have dimensions of 12.5 mm × 20 mm and are separated by a 0.2 mm gap to maintain proper impedance matching. Figure 2 illustrates the detailed geometry of the antenna along with the dimensions of every component. The proposed CPW antenna is designed to operate at the 2.45 GHz resonance frequency within the industrial, scientific, and medical (ISM) band, which makes the antenna suitable for wireless sensing, biomedical devices, and IoT-based communication systems. Numerical evaluation reveals a return loss of −63.33 dB at resonance, demonstrating excellent impedance compatibility between the feed structure and the radiating element.

This antenna serves as a benchmark design for evaluating the effects of mechanical deformation on its electromagnetic performance. Specifically, the aim is to analyze how bending influences the resonance frequency, reflection coefficient and overall radiation characteristics. The findings will facilitate the analysis of the difference between conventional bending and neutral axis bending of the antenna. Section 2.4 provides a detailed discussion on the encapsulation of the CPW antenna and the process of finding the neutral axis.

### 2.4. Locating the Neutral Axis and Encapsulation

**Figure 3:**
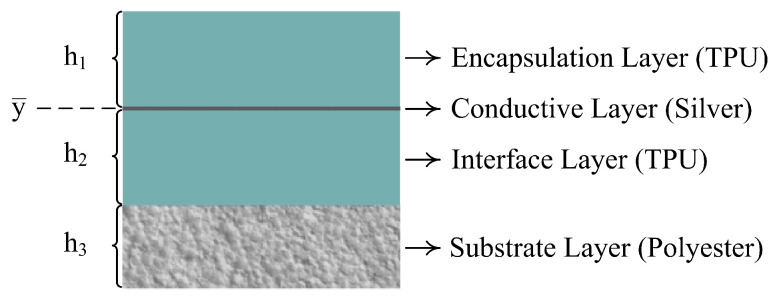
Cross-sectional structure of the composite CPW patch antenna and neutral axis of the multilayer beam.

Two more layers, the interface layer and the encapsulation layer, have been added as part of the encapsulation process to improve the mechanical robustness and bending performance of the proposed CPW antenna. These layers are designed to align the neutral axis with the conductive layer, thereby minimizing mechanical strain on the radiating element during deformation. Both layers are designed with TPU as the material. TPU is a flexible and durable material suitable for wearable and conformal antennas. As illustrated in Figure 3, the interface layer, positioned directly below the conductive layer, serves to reduce surface roughness and mitigate mechanical stress concentrations during bending [29]. The encapsulation layer, which encloses the entire antenna structure, is designed to provide additional structural stability and environmental protection. The thickness of the encapsulation layer is denoted by $h_{1}$, and the thickness of the interface layer is denoted by $h_{2}$. The position of the neutral axis is denoted with $\bar{y}$. The thicknesses of the encapsulation and interface layers are determined based on the position of the neutral axis to ensure $\bar{y}$ lies between these two layers. Once the neutral axis is located, the conductive layer is positioned so that the neutral axis aligns with the conductive layer.

Taking into consideration the elastic modulus of the different materials that make up the n-layered composite in Figure 3, the resultant force acting across the cross-section of the beam in pure bending about its neutral axis position can be expressed as [30]:

(11) $$F=\sum_{i}^{n}E_{i}x_{i}\int_{lower\;limit}^{upper\;limit}\frac{y}{R}dy=0$$

A tensile test is one of the common industrial techniques for measuring the elastic properties of materials [31]. It involves applying a controlled tension or force on a clamped material until it fails.

The lower and upper limits of the integral can be obtained for the $i^{th}$ layer as:

(12) $$\begin{array}{cc} lower\;limit & =\bar{y}-\sum_{j=1}^{i-1}h_{j}\\ upper\;limit & =\bar{y}-\sum_{j=1}^{i}h_{j} \end{array}$$

Hence, Equation (11) can be reduced as:

(13) $$\begin{array}{c} \sum_{i}^{n}E_{i}x_{i}\int_{\bar{y}-\sum_{j=1}^{i-1}h_{j}}^{\bar{y}-\sum_{j=1}^{i}h_{j}}\frac{y}{R}dy=0\\ \sum_{i}^{n}E_{i}x_{i}{\left[\frac{y^{2}}{2}\right]}_{\bar{y}-\sum_{j=1}^{i-1}h_{j}}^{\bar{y}-\sum_{j=1}^{i}h_{j}}=0 \end{array}$$

(14) $$\sum_{i}^{n}E_{i}x_{i}\left[{\left(\bar{y}-\sum_{j=1}^{i}h_{j}\right)}^{2}-{\left(\bar{y}-\sum_{j=1}^{i-1}h_{j}\right)}^{2}\right]=0$$

Applying the difference of two squares, then

(15) $$\sum_{i}^{n}E_{i}x_{i}\left[\left(2\bar{y}-\sum_{j=1}^{i-1}h_{j}-\sum_{j=1}^{i}h_{j}\right)\left(\sum_{j=1}^{i-1}h_{j}-\sum_{j=1}^{i}h_{j}\right)\right]=0$$

But,

(16) $$\sum_{j=1}^{i-1}h_{j}-\sum_{j=1}^{i}h_{j}=\;\left(h_{1}+h_{2}+\ldots +h_{i-1}\right)-\left(h_{1}+h_{2}+\ldots +h_{i-1}+h_{i}\right)=\;-h_{i}$$

(17) $$\sum_{i}^{n}E_{i}x_{i}\left[\left(2\bar{y}-\sum_{j=1}^{i-1}h_{j}-\sum_{j=1}^{i}h_{j}\right)\left(-h_{i}\right)\right]=0$$

(18) $$\sum_{i}^{n}E_{i}x_{i}\left[-2\bar{y}h_{i}+h_{i}\left(\sum_{j=1}^{i-1}h_{j}+\sum_{j=1}^{i}h_{j}\right)\right]=0$$

Also,

(19) $$\sum_{j=1}^{i-1}h_{j}+\sum_{j=1}^{i}h_{j}=\;\left(h_{1}+h_{2}+\ldots +h_{i-1}\right)-\left(h_{1}+h_{2}+\ldots +h_{i-1}+h_{i}\right)=\;2\sum_{j=1}^{i-1}h_{j}+h_{i}=\;2\sum_{j=1}^{i}h_{j}-h_{i}$$

(20) $$\sum_{i}^{n}E_{i}x_{i}\left[-2\bar{y}h_{i}+h_{i}\left(2\sum_{j=1}^{i}h_{j}-h_{i}\right)\right]=0$$

(21) $$\sum_{i}^{n}E_{i}x_{i}\left[2\bar{y}h_{i}-2h_{i}\sum_{j=1}^{i}h_{j}+h_{i}^{2}\right]=0$$

(22) $$2\sum_{i}^{n}E_{i}x_{i}h_{i}\bar{y}=\sum_{i}^{n}E_{i}x_{i}h_{i}\left(2\sum_{j=1}^{i}h_{j}-h_{i}\right)$$

The composite CPW patch antenna can be approximated as a multilayer beam shown in Figure 3 whose NA, $\bar{y}$, is the conductive layer. It is chosen such that the position of other layers in the beam is measured relative to it. Referring to Ballas’s model on piezoelectric beams [32], the conductive layer $\bar{y}$ can be obtained using,

(23) $$\bar{y}=\frac{\sum_{i=1}^{n}E_{i}x_{i}h_{i}\left(2\sum_{j=1}^{i}h_{j}-h_{i}\right)}{2\sum_{i=1}^{n}E_{i}x_{i}h_{i}}$$

where $E_{i}$ represents elastic modulus, $x_{i}$ represents widths, and $h_{i}$ represents the thickness of the beam’s $i^{th}$ layer.

**Figure 4:**
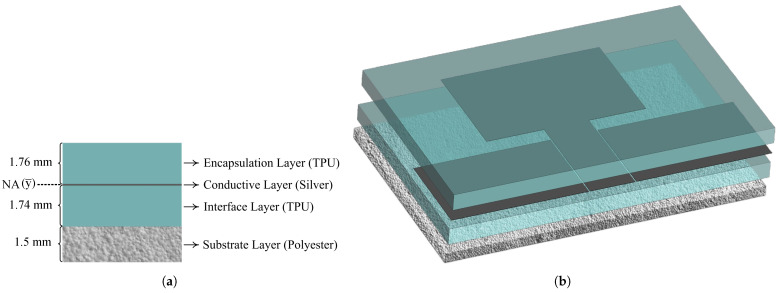
The composite shape of the encapsulated CPW patch antenna. (**a**) Calculated position of layers. (**b**) Encapsulated antenna.

Positioning the conductive layer of the antenna close to the neutral axis should optimize the performance of the antenna when being exposed to external stress such as bending or twisting and prevent any degradation in the performance of the antenna. The conductive layer has been empirically positioned by controlling the thickness of the encapsulation and interface layers, which aligns the conductive layer with the neutral axis. Figure 4 presents the composite shape of the encapsulated antenna, where the calculated position of each layer within the multilayer beam has been presented in Figure 4a with the ideal position of the conductive layer, and the multilayer structure of the antenna has been presented in Figure 4b.

### 2.5. Bending Configuration

To evaluate the performance of the antenna under mechanical deformation, the structure was bent along its length into a circular arc. The bending angle θ is defined as the central angle subtended by the arc of the bent antenna, measured with respect to the radius of curvature *R*. For an antenna of length *L* bent uniformly into a circular arc, θ and *R* are related by

(24) $$\theta =\frac{L}{R}\;\mathrm{or}\;\mathrm{equivalently}\;R=\frac{L}{\theta }$$

where θ is expressed in radians.

In this study, concave bending refers to the configuration in which the radiating patch (conductive layer) faces the inner surface of the bent arc, subjecting the conductive layer to compressive strain; convex bending refers to the configuration in which the patch faces the outer surface of the arc, subjecting the conductive layer to tensile strain. The CPW antenna analyzed in this study has a length of *L* = 47 mm, and using Equation (24), the antenna was simulated at bending angles of 15°, 30°, 60°, 90°, and 120°, corresponding to bending radii of 179.5, 89.8, 44.9, 29.9, and 22.4 mm, respectively, as summarized in Table 3.

## 3. Results and Discussion

### 3.1. Reflection Coefficient of the CPW Antenna

**Figure 5:**
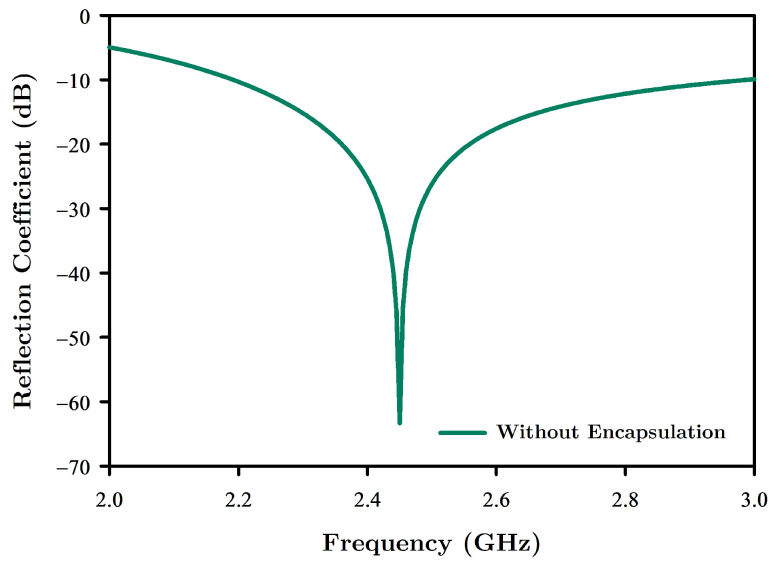
Reflection coefficient of the non-encapsulated CPW antenna.

The CPW antenna in this study serves as a reference design and has been optimized to operate within the ISM band at 2.45 GHz resonance frequency. Numerical evaluation reveals a return loss of −63.33 dB at resonance, demonstrating excellent impedance compatibility between the feed structure and the radiating element. The antenna also exhibits a frequency span of 2.19–2.98 GHz, enabling consistent performance throughout the intended operating band. Figure 5 presents the corresponding simulated results for reflection coefficients.

To comprehensively evaluate the antenna’s mechanical and electromagnetic behavior under deformation, a bending analysis has been conducted. The non-encapsulated CPW antenna has been simulated under varying bending angles ranging from 15° to 120°, and Section 3.1 provides a detailed discussion of these results.

#### Reflection Coefficient of the CPW Antenna in Bending Conditions

**Figure 6:**
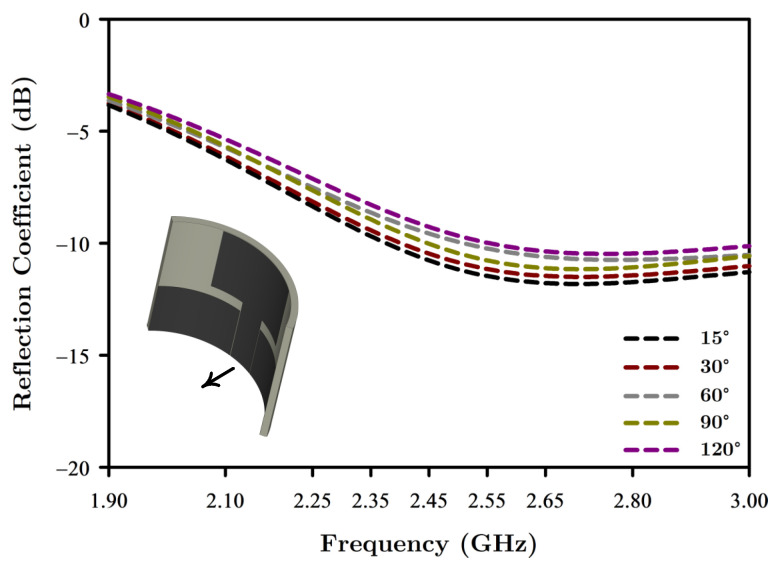
Reflection coefficient of the non-encapsulated CPW antenna (concave bending).

This section analyses the effects of mechanical stress on the electromagnetic performance of the flexible CPW antenna at various bending angles. The primary goal is to evaluate the influence of bending on key performance parameters, including resonance frequency shift, degradation of the reflection coefficient, and impedance stability. Figure 6 illustrates the simulated reflection coefficient of the CPW antenna in the concave bending condition at different bending angles: 15°, 30°, 60°, 90°, and 120°. The baseline performance, as shown in Figure 5, indicates that the antenna exhibits an optimal reflection coefficient of −63.33 dB when in a flat, unbent condition. However, as the antenna undergoes bending, its performance deteriorates significantly across all tested bending angles. The degradation in the reflection coefficient suggests increased impedance mismatch, resulting in poor return losses and reduced efficiency. The maximum reflection coefficient recorded under bending conditions is −11.8 dB, indicating a substantial reduction in performance. This behavior is consistent with the bending convention defined in Section 2.5: concave bending subjects the conductive layer to compressive strain, which is more likely to induce microcracking and localized conductivity degradation in the thin metallic layer than the tensile strain experienced under convex bending. These findings highlight the susceptibility of CPW antenna to mechanical deformation.

**Figure 7:**
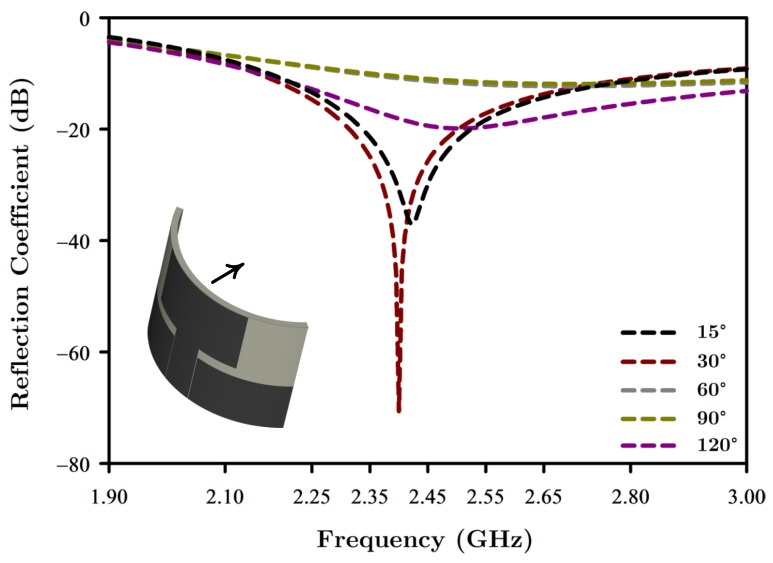
Reflection coefficient of the non-encapsulated CPW antenna (convex bending).

Figure 7 presents the simulated reflection coefficient of the CPW antenna in convex bending condition at different bending angles: 15°, 30°, 60°, 90°, and 120°. The results demonstrate a significant variation in antenna performance based on the bending angle, which highlights the impact of mechanical deformation on impedance matching and resonance stability.

The antenna exhibits significantly degraded resonance performance across all tested angles in the concave bending condition as referred to Figure 6. This indicates severe impedance mismatch and substantial performance degradation, and suggests that concave bending induces excessive mechanical strain on the conductive layer, disrupting the antenna’s electromagnetic properties and preventing the antenna from achieving stable resonance. Conversely, convex bending, which subjects the conductive layer to tensile strain, yields a relatively better response compared to the concave bending, however with inconsist response across different tested angles. At a 15° bending angle, the antenna maintains a strong resonance with a reflection coefficient of −36.9 dB. As the bending angle increases to 30°, the reflection coefficient improves significantly to −70.6 dB; however, a frequency shift is observed, with the resonance frequency shifting from 2.45 GHz to 2.4 GHz. Similarly, at 120°, the antenna achieves a reflection coefficient of −19.8 dB, but the resonance frequency shifts to 2.5 GHz. In contrast, at 60° and 90°, the antenna fails to maintain stable resonance performance, mirroring the poor performance observed in concave bending conditions.

For a flexible antenna, it is crucial to ensure optimal and stable performance under mechanical deformation. While this CPW antenna demonstrates good impedance matching and resonance retention under specific bending conditions, the overall performance of the antenna remains inconsistent across different bending angles. These findings emphasize the need for structural enhancements, such as material optimization, and encapsulation strategies, to mitigate performance degradation in bent or conformal applications. Section 3.2 explores the effectiveness of encapsulation in addressing these challenges and improving the antenna’s bending resilience.

### 3.2. Reflection Coefficient of the Encapsulated CPW Antenna

**Figure 8:**
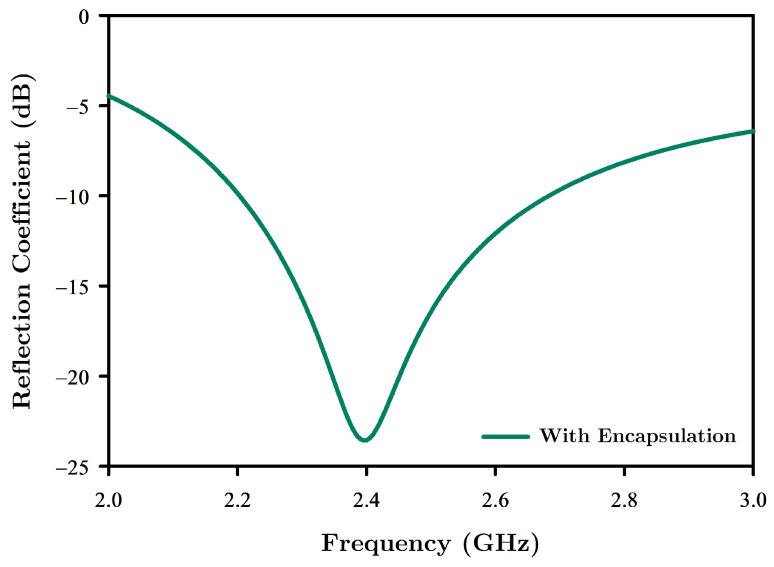
Reflection coefficient of the encapsulated CPW antenna.

The reflection coefficient of the encapsulated antenna has been illustrated in Figure 8, demonstrating the impact of encapsulation on the antenna’s performance. As observed, the encapsulated antenna resonates at 2.395 GHz, which still covers the intended applications’ frequency band, and the reflection coefficient at the 2.395 GHz resonance frequency has reduced to −23.58 dB. The encapsulation and interface layers surrounding the radiating patch introduce a dielectric loading effect, as these layers use TPU as material which is an electrically denser medium with properties listed in Table 2. This increases the effective permittivity of the antenna’s surrounding medium, and reduces the effective wavelength, resulting in a downward shift in the resonance frequency. Moreover, the altered effective permittivity modifies the impedance matching of the antenna, resulting in a reduction in the reflection coefficient. Furthermore, the operating bandwidth has also slightly decreased, now covering the range of 2.19 GHz to 2.67 GHz. Despite this reduction in bandwidth, the encapsulation plays a crucial role in enhancing the antenna’s mechanical durability, particularly in bent or deformed conditions. These layers help to maintain stable electromagnetic performance, ensuring the antenna remains functional in flexible and wearable applications where mechanical stress is inevitable. Section 3.2 discusses the performance of the encapsulated antenna during bending.

#### Reflection Coefficient of the Encapsulated CPW Antenna in Bending Conditions

**Figure 9:**
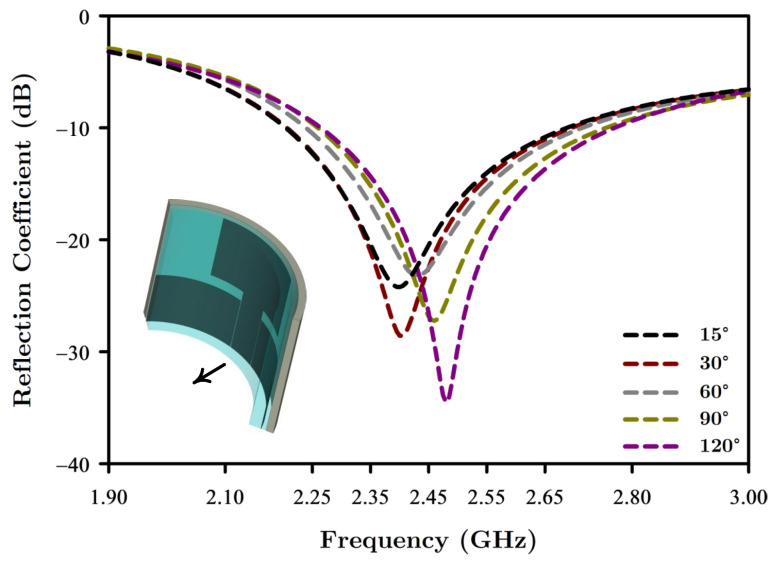
Reflection coefficient of the encapsulated CPW Antenna (concave bending).

This section analyses the effects of mechanical stress on the electromagnetic performance of the encapsulated CPW antenna at various bending angles similar to the non-encapsulated antenna. Figure 9 presents the simulated reflection coefficient of the encapsulated CPW antenna in the concave bending condition at different bending angles: 15°, 30°, 60°, 90°, and 120°. The encapsulation, composed of TPU, has been integrated into the antenna design to enhance mechanical durability, structural stability, and performance retention under bending conditions. The results indicate that the encapsulation has significantly improved the antenna’s resilience to bending-induced performance degradation. Unlike the non-encapsulated antenna, which exhibited complete resonance failure under concave bending, the encapsulated antenna retains its resonance characteristics across all bending angles. However, a shift in resonance frequency is observed, along with minor variations in the reflection coefficient at different angles. At a 15° bending angle, the antenna maintains a strong resonance at approximately 2.4 GHz, with a reflection coefficient of −24.2 dB. As the bending angle increases to 30°, the resonance shifts slightly; however, still approximately around 2.4 GHz, achieving an improved reflection coefficient of approximately −28.6 dB. For higher bending angles (60°, 90°, and 120°), the resonance frequency shifts progressively towards 2.48 GHz, with the reflection coefficient remaining within −20 dB to −35 dB.

**Figure 10:**
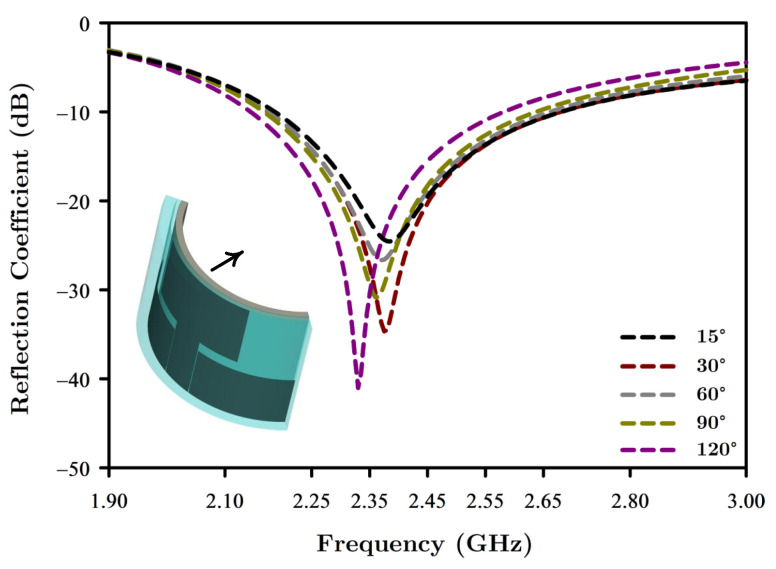
Reflection coefficient of the encapsulated CPW antenna (convex bending).

Figure 10 presents the simulated reflection coefficient of the encapsulated CPW antenna in convex bending condition at various angles: 15°, 30°, 60°, 90°, and 120°. The simulation results indicate that the antenna preserves its resonance characteristics under convex bending, with relatively stable impedance matching across the tested angles. At a 15° bending angle, the reflection coefficient reaches approximately −24.5 dB at 2.39 GHz, showing minimal performance degradation. When the bending angle increases to 30°, the impedance matching improves with a reflection coefficient of −34.6 dB. For bending angles of 60° and 90°, the resonance remains approximately at 2.37 GHz or close to this, with a reflection coefficient of around −30 dB. At 120°, the resonance shifts toward 2.33 GHz, with a reflection coefficient around −41 dB. 

### 3.3. Directivity of the CPW Antenna

**Figure 11:**
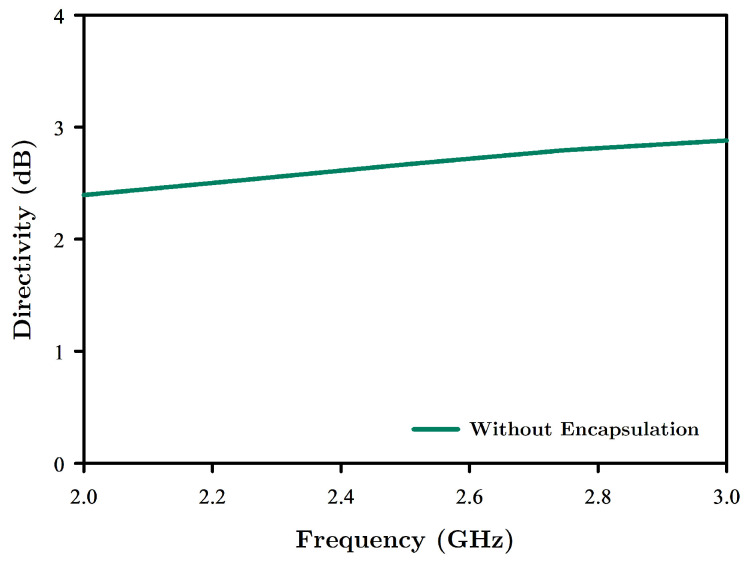
Directivity of the non-encapsulated CPW antenna.

The simulated directivity of the non-encapsulated CPW antenna is presented in Figure 11. The antenna exhibits a gradual increase in directivity across the operating frequency range, with a 2.79 dB peak in the operational band. The directivity is consistent with the near-omnidirectional radiation characteristics expected from a CPW-fed flexible antenna on an electrically thin substrate. The bending analysis of the non-encapsulated CPW antenna is discussed in Section 3.3.

#### Directivity of the CPW Antenna in Bending Conditions

**Figure 12:**
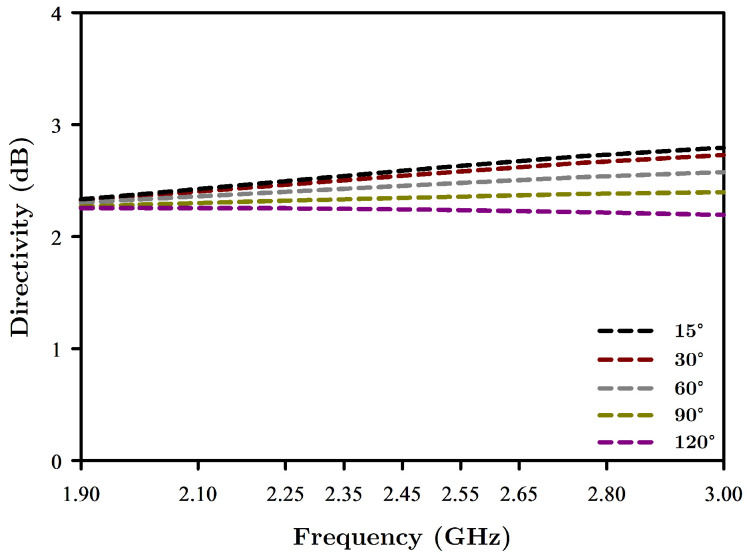
Directivity of the non-encapsulated CPW antenna (concave bending).

Figure 12 presents the simulated directivity of the non-encapsulated CPW antenna under concave bending conditions at bending angles of 15°, 30°, 60°, 90°, and 120°. The results indicate that the directivity starts to drift from around 2.1 GHz. Across all tested concave bending angles, the peak directivity in the band ranges from 2.22 dB to 2.71 dB. The results exhibit close grouping at lower frequencies, with separation becoming apparent toward the upper frequency range. The 120° concave bending condition yields the lowest directivity across the entire frequency range, with a notably flat response, remaining around 2.22 dB across the band, indicating that severe concave bending redistributes the radiated power and reduces the overall directivity of the antenna.

**Figure 13:**
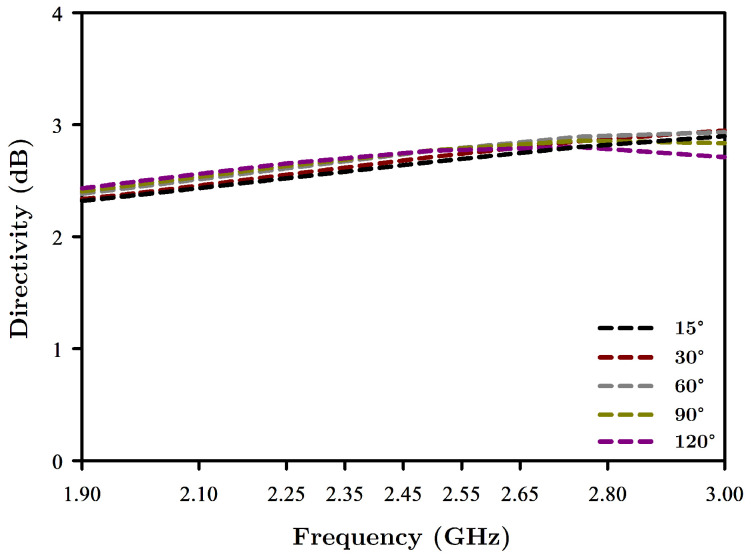
Directivity of the non-encapsulated CPW antenna (Convex Bending).

Figure 13 presents the simulated directivity of the non-encapsulated CPW antenna under convex bending conditions. In contrast to the concave bending results, convex bending introduces a stable directivity across different bending angles. At 15° and 30°, the directivity closely follows the flat antenna response, reaching a peak of around 2.80 dB in the operational band. This suggests that convex bending has a relatively limited influence on the directivity of the non-encapsulated antenna across the tested angles.

### 3.4. Directivity of the Encapsulated CPW Antenna

**Figure 14:**
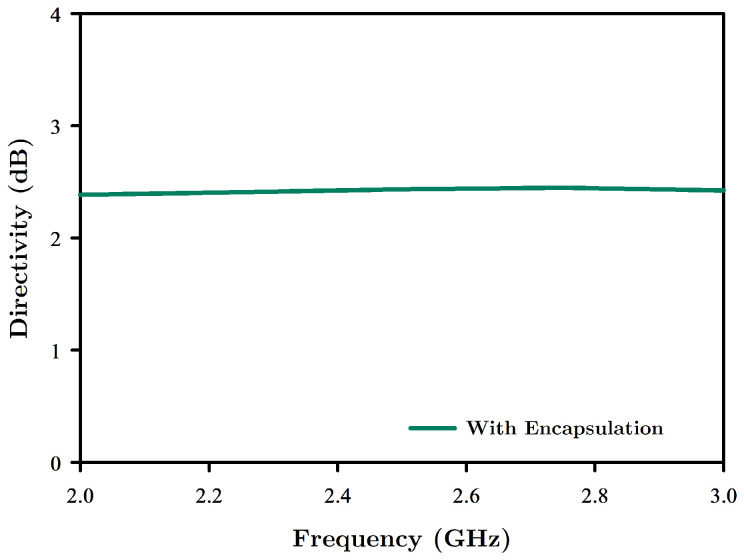
Directivity of the encapsulated CPW antenna.

The simulated directivity of the encapsulated CPW antenna is presented in Figure 14. In contrast to the non-encapsulated antenna, the encapsulated antenna exhibits a remarkably stable directivity across the entire operating frequency range, maintaining a directivity of approximately 2.40 dB to 2.45 dB throughout the operational band. This near-flat directivity response is attributed to the dielectric loading effect of the TPU encapsulation, which constrains the variation in radiation characteristics across frequency. The bending analysis of the encapsulated CPW antenna is discussed in Section 3.4.

#### Directivity of the Encapsulated CPW Antenna in Bending Conditions

**Figure 15:**
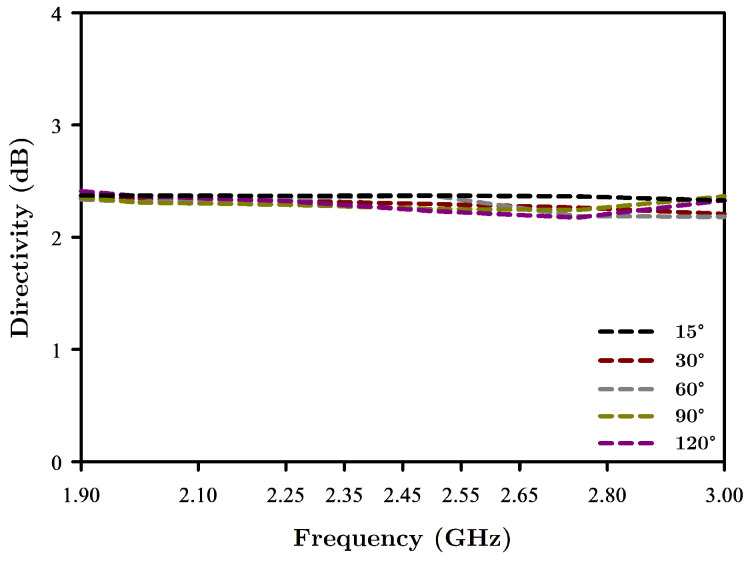
Directivity of the encapsulated CPW antenna (concave bending).

Figure 15 presents the simulated directivity of the encapsulated CPW antenna under concave bending conditions at bending angles of 15°, 30°, 60°, 90°, and 120°. The results demonstrate that the encapsulated antenna maintains relatively stable directivity across all tested concave bending angles, with a directivity of around 2.38 dB across the frequency range, representing notably tighter clustering of directivity results compared to the non-encapsulated antenna under equivalent concave bending conditions. The 120° bending angle exhibits a minor reduction in directivity, reaching approximately 2.19 dB at 2.75 GHz. This demonstrates that the TPU encapsulation significantly stabilizes the directivity of the antenna under bending, mitigating the pronounced angular dependence observed in the non-encapsulated bending conditions. 

**Figure 16:**
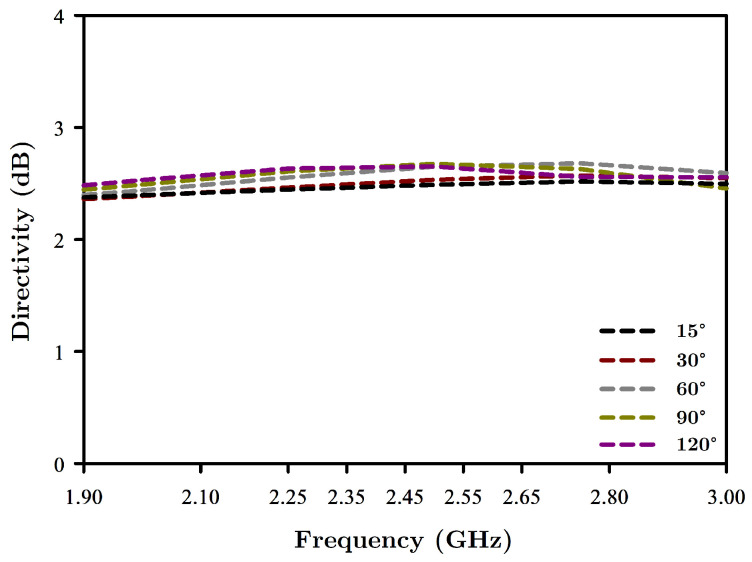
Directivity of the encapsulated CPW antenna (Convex Bending).

Figure 16 presents the simulated directivity of the encapsulated CPW antenna under convex bending conditions. A slight spread among the results is observed toward the mid-frequency range; however, all bending angles yield directivity within the range of approximately 2.48 dB to 2.67 dB, with a maximum spread of less than 0.2 dB. Nevertheless, the overall variation remains small, indicating that the encapsulation effectively preserves the directivity characteristics of the antenna under convex bending conditions.

### 3.5. Efficiency of the CPW Antenna

**Figure 17:**
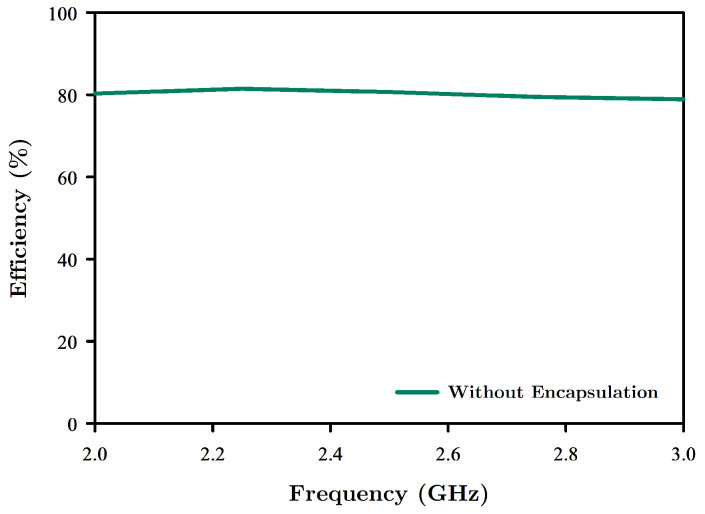
Efficiency of the non-encapsulated CPW antenna.

The simulated radiation efficiency of the non-encapsulated CPW antenna is presented in Figure 17. The antenna demonstrates consistently high efficiency across the operating frequency range, with a peak of 81.48% at 2.25 GHz and maintaining above 78.91% throughout the operational band. The relatively flat efficiency response indicates stable radiation performance across the intended ISM band. The bending analysis of the non-encapsulated CPW antenna efficiency is discussed in Section 3.5.

#### Efficiency of the CPW Antenna in Bending Conditions

**Figure 18:**
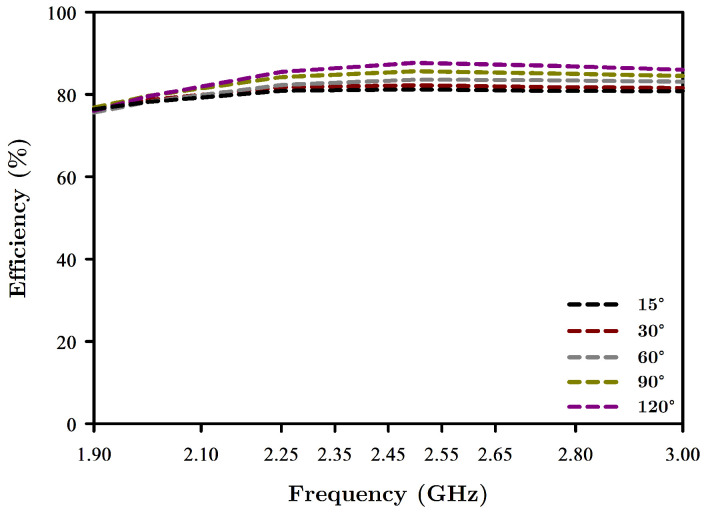
Efficiency of the non-encapsulated CPW antenna (concave bending).

Figure 18 presents the simulated radiation efficiency of the non-encapsulated CPW antenna under concave bending conditions at bending angles of 15°, 30°, 60°, 90°, and 120°. The results indicate that the efficiency remains relatively stable across all tested concave bending angles, with consistently maintained peak efficiencies within the range of 78.18% to 87.71%. A gradual increase in efficiency with increasing bending angle is observed, particularly at higher frequencies, where the 120° bending angle yields the highest efficiency of 87.71% at 2.5 GHz. Despite the significant impedance mismatch observed in the reflection coefficient under concave bending, the radiation efficiency remains largely unaffected, suggesting that the power radiated relative to the accepted power is preserved under mechanical deformation.

**Figure 19:**
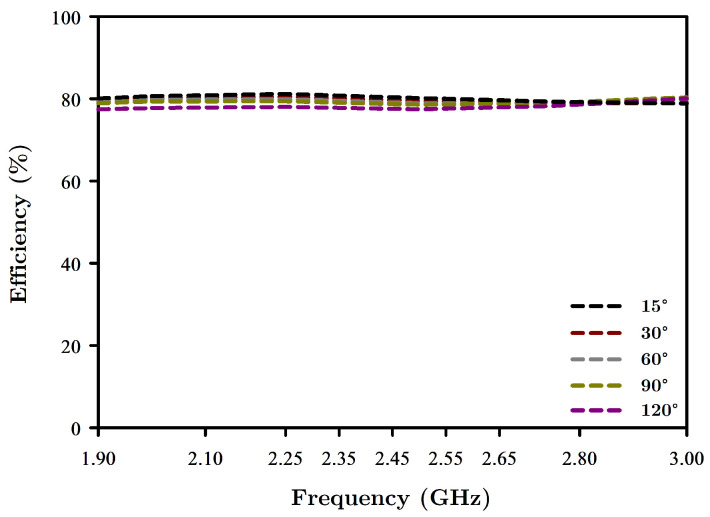
Efficiency of the non-encapsulated CPW antenna (convex bending).

Figure 19 presents the simulated radiation efficiency of the non-encapsulated CPW antenna under convex bending conditions. In contrast to the concave bending results, the convex bending efficiency results exhibit a tighter grouping across all bending angles, ranging from 77.78% to 81.22%. The results remain closely clustered throughout the operating band, with minimal angular dependence observed. These results demonstrate that convex bending has a negligible influence on the radiation efficiency of the non-encapsulated CPW antenna, with all configurations maintaining peak efficiency above 77%.

### 3.6. Efficiency of the Encapsulated CPW Antenna

**Figure 20:**
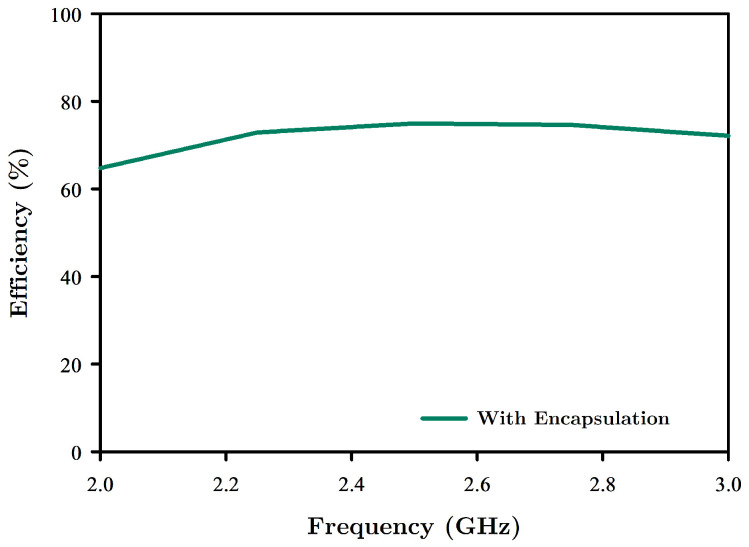
Efficiency of the encapsulated CPW antenna.

The simulated radiation efficiency of the encapsulated CPW antenna is presented in Figure 20. The encapsulation introduces a moderate reduction in radiation efficiency compared to the non-encapsulated antenna, with the encapsulated antenna exhibiting a peak efficiency of 74.97% at 2.5 GHz. This reduction in efficiency relative to the non-encapsulated antenna is attributed to the dielectric losses introduced by the TPU encapsulation and interface layers, which absorb a portion of the radiated power. Nevertheless, the encapsulated antenna maintains acceptable radiation efficiency across the entire operating band, and the bending analysis is discussed in Section 3.6.

#### Efficiency of the Encapsulated CPW Antenna in Bending Conditions

**Figure 21:**
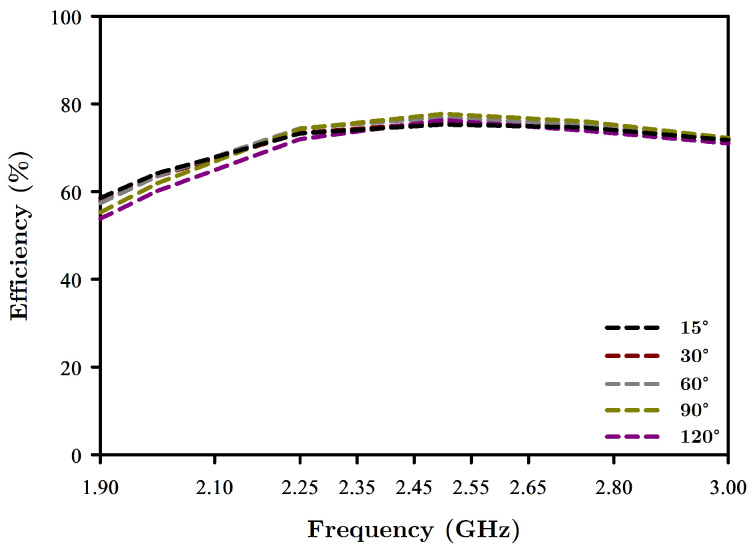
Efficiency of the encapsulated CPW antenna (concave bending).

Figure 21 presents the simulated radiation efficiency of the encapsulated CPW antenna under concave bending conditions at bending angles of 15°, 30°, 60°, 90°, and 120°. The results demonstrate that all bending configurations exhibit a similar trend to the flat encapsulated antenna, with efficiency rising from approximately 70% to a peak in the range of 72% to 78% near 2.5 GHz. A gradual increase in peak efficiency with increasing bending angle is observed, with the 90° concave bending configuration yielding the highest efficiency of 77.76% at 2.5 GHz among the concave bending conditions. The curves remain closely grouped throughout the frequency range, indicating that concave bending has a limited influence on the radiation efficiency of the encapsulated antenna.

**Figure 22:**
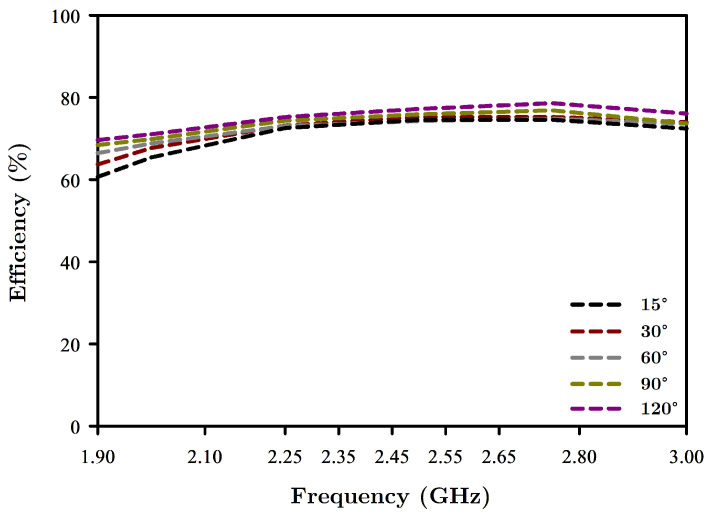
Efficiency of the encapsulated CPW antenna (Convex Bending).

Figure 22 presents the simulated radiation efficiency of the encapsulated CPW antenna under convex bending conditions. The convex bending results exhibit a broader spread among the curves compared to concave bending. However, as the frequency increases toward 2.5 GHz, the results converge, with all bending angles achieving efficiencies within the range of 74% to 78%. The 120° convex bending configuration yields the highest efficiency of 78.64% at 2.75 GHz. Overall, the encapsulated antenna demonstrates acceptable radiation efficiency retention under both concave and convex bending conditions across the operating band.

### 3.7. Comparison of Non-Encapsulated and Encapsulated Antenna Performance

Table 4 presents the variations in resonance frequency and reflection coefficient of the non-encapsulated and encapsulated antennas for different bending angles under both concave and convex bending conditions, enabling a direct comparison of their electromechanical stability. In comparison to the non-encapsulated antenna, the encapsulated design demonstrates greater stability in resonance frequency. The encapsulation effectively minimizes the impact of mechanical deformation, ensuring that the antenna maintains functional performance under bending conditions with negligible shifts in frequency, demonstrating the effect of neutral axis bending. Moreover, the variations in directivity and efficiency have also been presented in Table 4. While the non-encapsulated CPW antenna exhibits relatively high radiation efficiency and comparable directivity, these favorable radiation characteristics are rendered ineffective in practice due to the severe impedance mismatch introduced by mechanical deformation. As demonstrated in Section 3.1, the non-encapsulated antenna fails to maintain stable resonance across several bending angles. Consequently, despite the retained efficiency and directivity, the antenna is unable to effectively receive or transmit power under these conditions, as the impedance mismatch prevents adequate power transfer to the radiating element. In contrast, the encapsulated antenna maintains stable resonance across all tested bending angles under both concave and convex conditions, albeit at a moderate reduction in radiation efficiency. A comprehensive analysis of the impact of neutral axis bending on antenna performance has been provided in Section 3.8.

### 3.8. Analysis of the Effect of Neutral Axis

The position of the conductive layer relative to the neutral axis plays a critical role in determining the impedance mismatch due to mechanical strain experienced by the antenna during bending, and consequently, performance stability of the antenna. As illustrated in Figure 3, by adjusting $h_{1}$ and $h_{2}$ together while keeping the total beam height constant, the conductive layer can be intentionally shifted to deviate from the neutral axis. To observe the effect of neutral axis, the thickness of the encapsulation layer and the interface layer has been varied while keeping the total thickness of the antenna constant.

**Figure 23:**
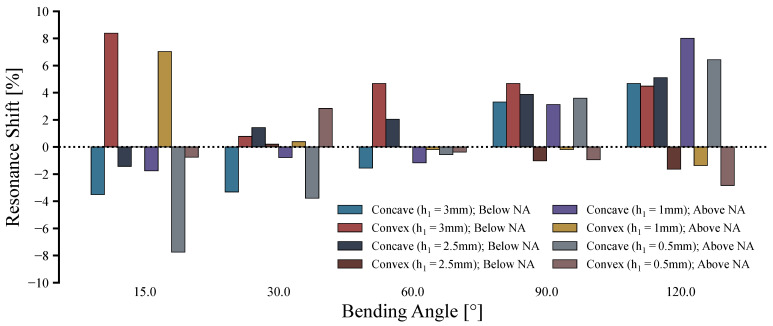
Shift in resonance frequency of encapsulated antenna when the conductive layer is displaced from neutral axis.

When $h_{1}$ = 3 mm or 2.5 mm, the conductive layer is positioned below the neutral axis. In this configuration, concave bending places the conductive layer under tensile strain, while convex bending induces compressive strain. On the other hand, when $h_{1}$ = 1 mm or 0.5 mm, the conductive layer is positioned above the neutral axis, and in this configuration, concave bending places the conductive layer under compressive strain, while convex bending induces tensile strain. This mechanical asymmetry is clearly reflected in the resonance frequency shifts in the antenna as presented in Figure 23. Significant and non-uniform resonance frequency deviations can be observed, particularly under concave bending. The frequency shifts are distributed in both lower and higher frequency directions, ranging from −7.75% to 8.38% for various bending angles from 15° to 120°. The maximum shift in resonance frequency can be observed when $h_{1}$ = 3 mm, where the frequency shift ranges from −3.51% to 8.38%. For $h_{1}$ = 2.5 mm, the frequency shift varies between −1.63% to 5.1%. Similarly, for $h_{1}$ = 1 mm, the antenna exhibits frequency shifts ranging from −1.76% to 8.01%. For $h_{1}$ = 0.5 mm, the antenna shows the maximum negative shift, with shifts ranging from −7.75% to 6.43%.

**Figure 24:**
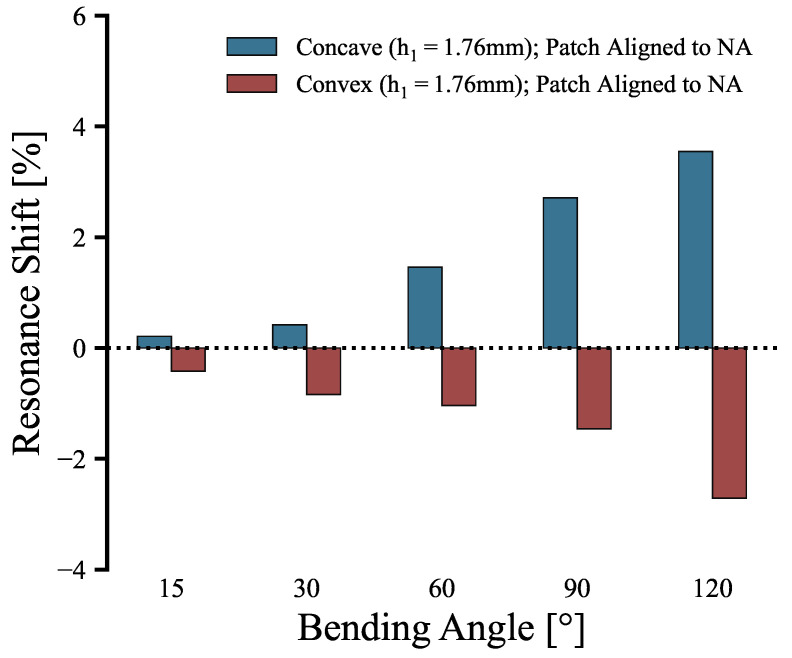
Shift in resonance frequency of encapsulated antenna when the conductive layer is aligned with neutral axis.

When $h_{1}$ is set to 1.76 mm, the conductive layer is geometrically aligned with the neutral axis $\bar{y}$. Under this condition, the conductive layer lies at the plane of zero strain, and as a result, both concave and convex bending impose negligible deformation on the radiating element. The results presented in Figure 24 confirm this, where it can be observed that the resonance shift remains confined within a narrow range of approximately −2.71% to 3.55% across all tested bending angles from 15° to 120°. Furthermore, the shift in resonance is very uniform and increases with increasing bending angles. While in the concave bending condition, the resonance frequency is shifted to the right with progressive increase in magnitude with increasing bending angle; whereas convex bending shifts the resonance frequency to the left with progressive increase in magnitude with increasing bending angle. These results demonstrate that positioning the conductive layer at the neutral axis effectively decouples the antenna’s electrical response from mechanical deformation.

### 3.9. Radiation Pattern Analysis

The radiation pattern of an antenna is a crucial parameter that defines its ability to transmit or receive electromagnetic waves in specific directions. For flexible and wearable antennas, radiation characteristics can be significantly influenced by mechanical deformations such as bending, twisting, and stretching [33]. Therefore, a comprehensive analysis of the radiation pattern under different bending conditions is essential to evaluate the antenna’s suitability for real-world applications. The 2D radiation pattern is useful for quantifying directional metrics such as the half-power beamwidth (HPBW) and directivity along principal planes, while the 3D radiation pattern is highly effective for visualizing the overall radiation characteristics of the antenna, including beamwidth, directivity, and null regions [34].

#### 3.9.1. Radiation Pattern of CPW Antenna and Encapsulated CPW Antenna

**Figure 25:**
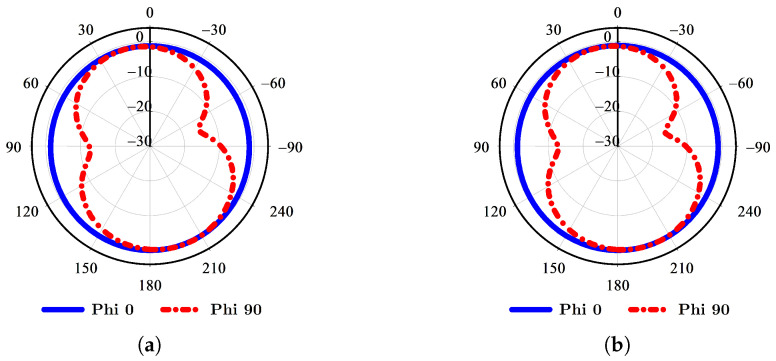
2D radiation pattern of proposed antenna. (**a**) CPW antenna. (**b**) Encapsulated CPW antenna.

Figure 25 presents the 2D radiation patterns for Phi $=0^{\circ }$ and Phi $=90^{\circ }$ of the CPW antenna in both non-encapsulated and encapsulated configurations. Figure 25a illustrates the 2D radiation pattern of the non-encapsulated antenna, while Figure 25b illustrates the 2D pattern of the encapsulated antenna. For the non-encapsulated CPW antenna, the HPBW is 100° with a directivity of 2.8 dB. For the encapsulated antenna, the HPBW is 97.5° with a directivity of 2.45 dB.

**Figure 26:**
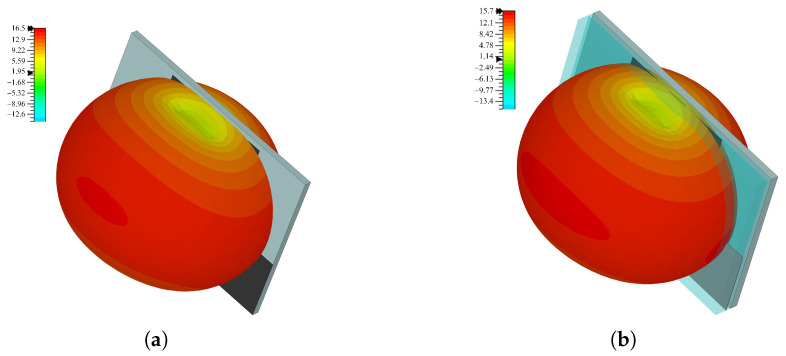
Radiation pattern of proposed antenna. (**a**) CPW antenna. (**b**) Encapsulated CPW antenna.

Figure 26 presents the corresponding 3D radiation patterns of the CPW antenna in both configurations, offering a comprehensive visualization of the radiation characteristics. Before encapsulation, the CPW antenna achieves a peak radiation intensity of 16.5 dB(V/m). After the encapsulation process, the maximum radiation slightly decreases to 15.33 dB(V/m). This marginal reduction suggests that the encapsulation has minimal impact on the overall radiation power of the antenna, and does not significantly attenuate the emitted signal. The slight reduction in peak radiation intensity can be attributed to the presence of the encapsulation layer, which introduces a minor dielectric loading effect. However, the overall radiation pattern remains consistent, demonstrating that the antenna’s fundamental characteristics are well-preserved despite the additional encapsulation. Section 3.9.2 will discuss more on the radiation pattern of the encapsulated CPW antenna under deformation or mechanical stress.

#### 3.9.2. Radiation Pattern of Encapsulated CPW Antenna During Bending

**Figure 27:**
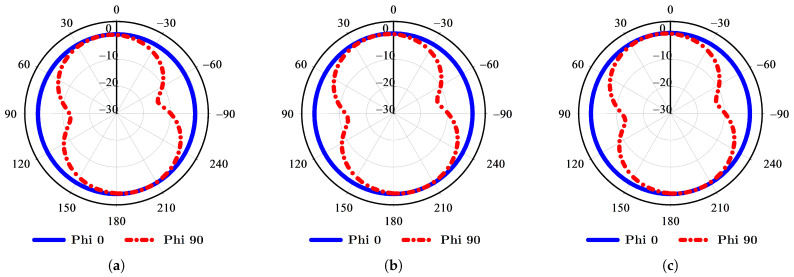
2D Radiation pattern of encapsulated CPW antenna during concave bending. (**a**) 30°. (**b**) 60°. (**c**) 90°.

Figure 27 presents the 2D radiation patterns of the encapsulated CPW antenna under concave bending at angles of 30°, 60°, and 90°. Specifically, Figure 27a–c illustrate the 2D radiation patterns for the 30°, 60°, and 90° bending angles, respectively. The corresponding HPBW are 97.5°, 95°, and 95°, with directivity of 2.32 dB, 2.37 dB, and 2.29 dB, respectively.

**Figure 28:**
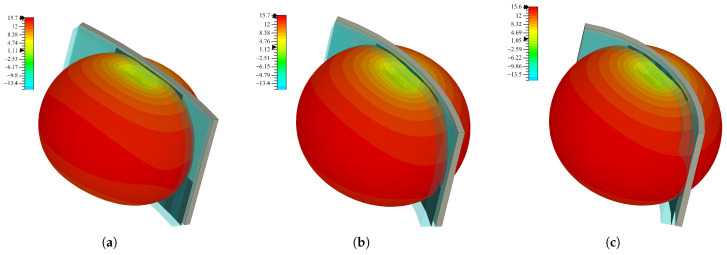
Radiation pattern of encapsulated CPW antenna during concave bending. (**a**) 30°. (**b**) 60°. (**c**) 90°.

Figure 28 presents the corresponding 3D radiation patterns of the encapsulated CPW antenna under concave bending at the same angles. The simulation results indicate that the radiation direction remains consistent across all bending angles, demonstrating the antenna’s ability to maintain its primary radiation characteristics despite mechanical deformation. Furthermore, the peak radiation intensities for 30°, 60°, and 90° bending conditions are recorded as 15.65 dB(V/m), 15.67 dB(V/m), and 15.60 dB(V/m), respectively. These results suggest a stable radiation performance, with only minimal variations in radiation power across different bending angles.

**Figure 29:**
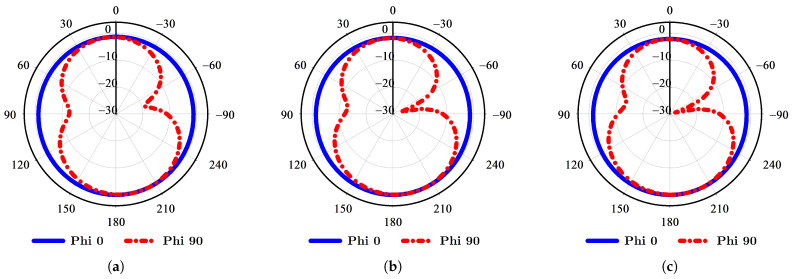
2D radiation pattern of encapsulated CPW antenna during convex bending. (**a**) 30°. (**b**) 60°. (**c**) 90°.

Similarly, Figure 29 presents the 2D radiation patterns of the encapsulated CPW antenna under convex bending at angles of 30°, 60°, and 90°. Specifically, Figure 29a–c represent the 2D patterns for the 30°, 60°, and 90° bending angles, respectively. The corresponding HPBW are 100°, 105°, and 110°, with directivity of 2.57 dB, 2.68 dB, and 2.68 dB, respectively. Compared to the concave bending, the beamwidth broadens progressively with increasing convex bending angle, while directivity increases slightly, reflecting the change in effective aperture shape under deformation.

**Figure 30:**
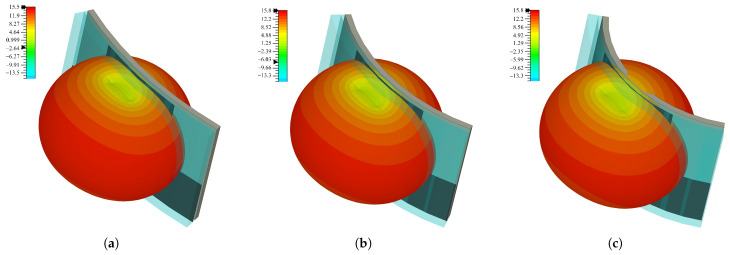
Radiation pattern of encapsulated CPW antenna during convex bending. (**a**) 30°. (**b**) 60°. (**c**) 90°.

Figure 30 illustrates the corresponding 3D radiation patterns of the encapsulated CPW antenna under convex bending at the same angles. Similar to the concave bending scenario, the antenna maintains a consistent radiation pattern across all convex bending conditions, indicating the structural and electromagnetic robustness of the antenna under mechanical stress. The recorded peak radiation intensities for 30°, 60°, and 90° bending conditions are 15.54 dB(V/m), 15.79 dB(V/m), and 15.83 dB(V/m), respectively. These results demonstrate that the encapsulated CPW antenna retains stable radiation performance even under convex bending conditions, with minimal fluctuations in radiation intensity.

The observed consistency in radiation characteristics, together with the moderate variation in HPBW and directivity across bending angles, highlights the structural resilience and electromagnetic stability of the encapsulated CPW antenna under mechanical stress. The results confirm that the encapsulation process effectively preserves radiation performance, making it well-suited for applications where deformation is expected during operation.

## 4. Comparative Analyses with Recent State-of-the-Art Literature

Results from recent state-of-the-art studies have been presented in Table 5. The authors in [27,35] presented compact flexible antennas with multiband capability, having electrical dimensions of only $0.23\lambda_{\circ }\times 0.17\lambda_{\circ }$ and $0.20\lambda_{\circ }\times 0.25\lambda_{\circ }$ respectively. However, despite achieving substantial miniaturization, the proposed antennas exhibited considerable resonance frequency shifts under mechanical deformation, with shifts reaching up to −11.76% and 16.46%. In addition, the bending analysis was restricted to maximum bending angles of 60° and 90°, respectively. Similarly, the antenna reported in [36] was evaluated under bending conditions with bending radii of up to 80 mm and exhibited a resonance frequency shift of up to −18.37%. Likewise, the antenna presented in [37] was tested for bending radii up to 130 mm and demonstrated resonance frequency variations of up to 11.84%. A relatively small resonance frequency shift of approximately −6% was reported in [38]; however, the antenna was evaluated only for limited bending radii of 10 mm and 20 mm. In addition, the bending analysis was restricted exclusively to convex deformation conditions. Similar limitations are also observed in [27,35,36,37], where the antenna performance was investigated only under convex bending conditions.

As observed in this study, the antenna exhibits the most significant performance degradation under concave bending conditions, where the conductive layer experiences compressive strain. Consequently, the resonance frequency shifts reported in the aforementioned studies could potentially increase under concave deformation conditions. In contrast, the proposed antenna, which uses the neutral axis phenomenon, has demonstrated performance stability during both concave and convex bending conditions with test angles up to 120° while maintaining resonance frequency shift under 3.55% for this extreme bending.

## 5. Conclusions

Design and deformation analysis of a flexible 2.45 GHz CPW patch antenna incorporating neutral axis-based encapsulation for improved electromagnetic stability has been presented in this study. The proposed antenna, with an overall electrical size of $0.25\lambda_{\circ }\times 0.38\lambda_{\circ }$, utilizes a polyester substrate, silver conductive layer, and TPU encapsulation.

Analysis has been conducted with a non-encapsulated reference antenna, a neutral axis-aligned encapsulated antenna, and an encapsulated antenna with the conductive layer intentionally displaced away from the neutral axis. The reference non-encapsulated antenna achieved a reflection coefficient of −63.33 dB at 2.45 GHz. After encapsulation, the antenna resonates at 2.4 GHz with a reflection coefficient of −23.58 dB, while still maintaining adequate impedance matching within the intended operating band for the targeted application. The results demonstrated that the non-encapsulated antenna suffered substantial resonance degradation under bending conditions, particularly for concave bending. Whereas the encapsulated antenna with the conductive layer aligned along the neutral axis maintained stable electromagnetic performance under both convex and concave bending conditions, sustaining bending angles reaching 120°. The proposed neutral axis-aligned configuration achieved a maximum resonance frequency shift of only 3.55%, significantly lower than the 8.38% shift observed when the conductive layer was displaced away from the neutral axis.

These findings validate the effectiveness of the neutral axis technique in minimizing deformation-induced strain and improving the electromagnetic stability of flexible antennas. However, the current study is limited to simulation-based analysis, and experimental validation remains an important direction for future work. In practical implementation, accurate alignment of the conductive layer with the neutral axis depends on precise control of the TPU encapsulation and interface layers thicknesses, as slight variations in layers thickness may alter the neutral axis position and affect antenna performance under deformation. Therefore, future investigations will focus on fabrication processes and precision thickness-control techniques for TPU encapsulation to enable accurate neutral axis implementation in practical wearable antenna systems.

## Figures and Tables

**Table 1:** Mesh convergence analysis for antenna configurations in normal and bending conditions.

Antenna	Mesh Cells	Resonance Frequency (GHz)	Step Deviation (GHz)
CPW	139,536	2.45	0.015
	114,700	2.435	0.025
	96,600	2.41	0.030
	69,741	2.38	
CPW (Bending)	567,000	2.505	0.010
	506,328	2.495	0.020
	462,000	2.475	0.020
	435,200	2.455	
Encapsulated CPW	255,000	2.395	0.000
	237,600	2.395	0.005
	199,584	2.39	0.010
	151,800	2.38	0.025
	119,712	2.355	
Encapsulated CPW (Bending)	452,088	2.48	0.005
	438,464	2.475	0.005
	398,208	2.47	0.010
	359,900	2.46	0.010
	330,600	2.45	

**Table 2:** Detailed list of materials used in the CST simulation.

Component	Material	Specification		Ref.
Conductive Patch	Silver	Conductivity (S/m)	$6.301\times 10^{7}$	[25]
		Young’s Modulus (kN/mm^2^)	76	
Substrate	Polyester	Dielectric Constant	1.5	[3]
		Young’s Modulus (kN/mm^2^)	3.25	
Encapsulation	Thermoplastic Polyurethane (TPU)	Dielectric Constant	3.5	[26]
		Young’s Modulus (kN/mm^2^)	0.033	

**Table 3:** Bending angles and corresponding bending radii.

***θ*** (°)	15	30	60	90	120
**Radii (mm)**	179.5	89.8	44.9	29.93	22.44

**Table 4:** Comparative performance analysis of the non-encapsulated and encapsulated antenna.

Bending Condition	Bending Angle	Non-Encapsulated CPW Antenna				Encapsulated CPW Antenna			
		**Resonance Frequency (GHz)**	**Reflection Coefficient (dB)**	**Directivity (dB)**	**Efficiency (%)**	**Resonance Frequency (GHz)**	**Reflection Coefficient (dB)**	**Directivity (dB)**	**Efficiency (%)**
Flat	—	2.45	−63.33	2.8	81.48	2.395	−23.58	2.45	74.97
Concave	15°	2.7	−11.82	2.72	81.24	2.4	−24.24	2.37	75.32
	30°	2.71	−11.51	2.66	82.24	2.405	−28.61	2.32	75.77
	60°	2.78	−10.74	2.53	83.62	2.43	−23.22	2.37	76.94
	90°	2.71	−11.15	2.38	85.63	2.46	−27.25	2.29	77.76
	120°	2.76	−10.47	2.25	87.71	2.48	−34.54	2.32	76.32
Convex	15°	2.43	−36.9	2.8	81.22	2.39	−24.55	2.52	74.60
	30°	2.4	−70.63	2.85	80.53	2.38	−34.67	2.57	75.31
	60°	2.68	−12.28	2.89	79.94	2.37	−26.64	2.68	74.88
	90°	2.7	−11.87	2.86	79.45	2.36	−30.84	2.68	76.90
	120°	2.51	−19.85	2.8	78.27	2.33	−41.04	2.65	78.64

**Table 5:** Comparison of the proposed antenna with recent state-of-the-art literature.

Ref.	Frequency (GHz)	Application	Material	Size ($\lambda^{2}$)	Direction	Bending	Resonance Shift (Max)
[37]	2.45	ISM	Jeans	$0.65\lambda_{\circ }\times 0.65\lambda_{\circ }$	Convex; Horizontal	r= 30, 50, 70, 130	11.84%
[27]	1.7, 2.4, 3.8, 4.6,5.2	ISM, LoRa, 5G	Felt	$0.23\lambda_{\circ }\times 0.17\lambda_{\circ }$	Convex; Vertical and Horizontal	θ= 20, 40, 60	−11.76%
[36]	2.45	ISM	Cotton cloth	$0.93\lambda_{\circ }\times 1.01\lambda_{\circ }$	Convex; Vertical and Horizontal	r= 40, 60, 80	−18.37%
[38]	4.25	ISM, 5G	PDMS	$0.35\lambda_{\circ }\times 0.35\lambda_{\circ }$	Convex; Vertical and Horizontal	r= 10, 20	−6%
[35]	1.4, 3.5, 5.6	5G, GPS	Polyimide	$0.20\lambda_{\circ }\times 0.25\lambda_{\circ }$	Convex; Vertical and Horizontal	θ= 45, 60, 90	16.46%
This Study	2.4	ISM	Polyester	$0.25\lambda_{\circ }\times 0.38\lambda_{\circ }$	Concave and Convex; Horizontal	θ= 15, 30, 60, 90, 120	3.55%

## Data Availability

The original contributions presented in this study are included in the article/Supplementary Materials. Further inquiries can be directed to the corresponding authors.

## Supplementary Materials

The following supporting information can be downloaded at: https://www.mdpi.com/article/10.3390/s26154876/s1, Table S1: Reflection coefficient (S11) data of the non-encapsulated CPW antenna including bending results (S11 Data.xlsx); Table S2: Reflection coefficient (S11) data of the encapsulated CPW antenna including bending results (S11 Data - Enc.xlsx); Table S3: Reflection coefficient (S11) data of the encapsulated CPW antenna with 0.5 mm encapsulation thickness including bending results (S11 Data - Enc 0.5mm.xlsx); Table S4: Reflection coefficient (S11) data of the encapsulated CPW antenna with 1 mm encapsulation thickness including bending results (S11 Data - Enc 1mm.xlsx); Table S5: Reflection coefficient (S11) data of the encapsulated CPW antenna with 2.5 mm encapsulation thickness including bending results (S11 Data - Enc 2.5mm.xlsx); Table S6: Reflection coefficient (S11) data of the encapsulated CPW antenna with 3 mm encapsulation thickness including bending results (S11 Data - Enc 3mm.xlsx); Table S7: Resonance frequency, reflection coefficient at resonance, and percentage shift in resonance frequency for all bending angles (Resonance Data.xlsx).
